# MXene Lubricated Tribovoltaic Nanogenerator with High Current Output and Long Lifetime

**DOI:** 10.1007/s40820-023-01198-z

**Published:** 2023-10-07

**Authors:** Wenyan Qiao, Linglin Zhou, Zhihao Zhao, Peiyuan Yang, Di Liu, Xiaoru Liu, Jiaqi Liu, Dongyang Liu, Zhong Lin Wang, Jie Wang

**Affiliations:** 1 Beijing Institute of Nanoenergy and Nanosystems, Chinese Academy of Sciences, Beijing, 100083 People’s Republic of China; 2 School of Nanoscience and Engineering, University of Chinese Academy of Sciences, Beijing, 100049 People’s Republic of China; 3 School of Materials Science and Engineering, Georgia Institute of Technology, Atlanta, GA 30332 USA

**Keywords:** Tribovoltaic nanogenerators, Ultra-robust, Interface wear, Interface lubricant

## Abstract

Tribovoltaic nanogenerators (TVNGs) have the characteristics of high current density, low matched impedance and continuous output, which is expected to solve the problem of power supply for small electronic devices. However, wear occurrence in friction interface will seriously reduce the performance of TVNGs as well as lifetime. Here, we employ MXene solution as lubricate to improve output current density and lifetime of TVNG simultaneously, where a high value of 754 mA m^−2^ accompanied with a record durability of 90,000 cycles were achieved. By comparing multiple liquid lubricates with different polarity, we show that conductive polar liquid with MXene as additive plays a crucial role in enhancing the electrical output performance and durability of TVNG. Moreover, the universality of MXene solution is well demonstrated in various TVNGs with Cu and *P*-type Si, and Cu and *N*-GaAs as material pairs. This work may guide and accelerates the practical application of TVNG in future.

**Supplementary Information:**

The online version contains supplementary material available at 10.1007/s40820-023-01198-z.

## Highlights

- Successfully solves the key issue of tribovoltaic nanogenerators (TVNGs) lifetime (90,000 cycles) and improves its output current density (754 mA m^−2^) simultaneously.
- Conductive polar liquid with MXene as additive is proposed as the dominant factor in enhancing the electrical output performance and durability of TVNG simultaneously.
- The mechanism of lubricated TVNG with enhanced output performance is explained from the perspective of solution polarity at the first time.
- Mxene solution exhibits universality in different types of semiconductor systems (Cu and *P*-type Si, and Cu and *N*-GaAs as material pairs).

**Supplementary Information:**

The online version contains supplementary material available at 10.1007/s40820-023-01198-z.

## Graphical abstract

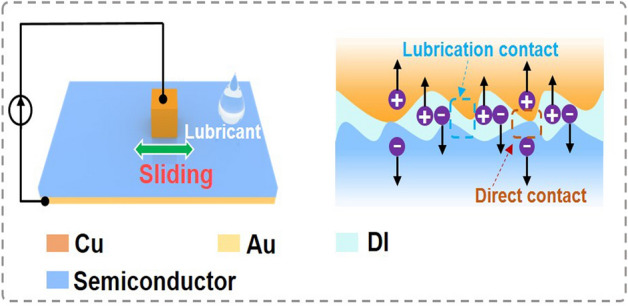

## Introduction

With the rapid development of sustainable renewable energy, a large number of portable electronic devices and distributed sensors are emerging [1–13]. At present, the majority of electronic devices and sensors require external battery supply, which increases the cost of equipment replacement, and also brings a huge ecological environmental burden [14]. Tribovoltaic nanogenerators (TVNGs) can directly generate direct current and collect energy directly from the environment to power small electronic devices and distributed sensors without any rectifier devices [15–39], promoting human society into the era of big data, artificial intelligence (AI) and the Internet of Things (IoTs) [32]. However, serious hard contact wear will lead to a rapid decline in the output performance of TVNG, which is an urgent problem to be solved for the practical application of TVNG.

Various methods have been proposed to solve this problem, such as optimizing friction pairs and interface lubrication. Recently, 4H-SiC and Cu were used as a friction pair to fabricate TVNG device, which has improved output current at high humidity and can maintain good stability after 400 cycles [33]. Instead of Cu with Mxene powder and Si as a friction pair, the current density and lifetime of TVNG increased to 97.8 mA m^−2^ and 1800 cycles [34]. For further improving the lifetime of TVNG, polyalphaolefin SpectraSyn 4 (PAO4) was added to the contact surface to achieve interface lubrication, and the lifetime of TVNG raised to 20,000 cycles, but the corresponding current density was only 30 mA m^−2^ [35]. To increase the current density and durability of TVNG simultaneously, water-based GO solution was proposed to lubricating TVNG in our previous work, and a record output current density of 775 mA m^−2^ accompanied with the lifetime of 30,000 cycles were realized [32], where the current density was 5.53 times higher than that of previous reported highest value 140 mA m^−2^ [39]. Although the output performance of TVNG have been elevated to a high record value, a better durability is still desirable to promote the practical applications of TVNG. Besides, according to previous reports, interface lubrication is a very effective way to prolong the lifetime of TVNG, but it does not always work for improving the output performance of TVNG. Hence, understanding the underlying relationships between lubricants and the mechanical/electrical properties of TVNG can provide a guideline for selecting appropriate liquid lubricants to designing high output performance TVNG with ideal lifetime.

In this work, we propose a MXene solution lubricated TVNG (MXene-TVNG), which can improve the current density and lifetime simultaneously. By using MXene solution as lubricant, a high current density of 754 mA m^−2^ with a record durability of 90,000 cycles are achieved at the first time for macro sliding mode metal semiconductor TVNG. Through investigating the influence of polar and electrical properties of different lubricants on the electrical output performance and durability of TVNG, we found that polar liquid lubricant with good conductivity can fill the interfacial gap, and then effectively reduce the interface dynamic resistance of TVNGs, resulting higher electron–hole pairs transfer efficiency. Combining with additional charges carriers induced by solid–liquid trobovoltic effect between polar liquid and *P*-type Si, the output current density of MXene-TVNG is 104.8 times and 314.6 times higher than that of original TVNG and oil-TVNG, respectively. Besides, two-dimensional material such as MXene as lubricant additive can largely reduce the mechanical wear at the interface, then greatly prolong the lifetime of TVNG. Thus MXene-TVNG also has 90% of initial current density after 90,000 cycles, which is a new record value for macro sliding mode metal semiconductor TVNG. Moreover, MXene solution exhibited the universality in various TVNGs with Cu and *P*-type Si, and Cu and *N*-GaAs as material pairs. This work reveals the key factors of liquid lubricants in TVNG, and guides the design of high performance TVNG through liquid interface lubrication.

## Experimental Section

### Fabrication of the TVNG

TVNG consists of a metal slider and semiconductor polishing wafer. The fabrication of the metal slider: (1) Select 3 mm thick acrylic as substrate material, and use laser cutting machine (PLS6.75 universal laser system) to cut out 5 mm × 5 mm small squares for use. (2) Cut 5 mm × 7 mm copper foil adhered to 5 mm × 5 mm acrylic substrate, and lead out 2 mm electrode connecting wire. The fabrication of the semiconductor friction polishing wafer: (1) The silicon wafers of *P*-type Si (1–20 Ω cm) was purchased from Zhejiang Lijing Photoelectric Technology Co., LTD, with a specification of 4 inches. The crystal was planar oriented along [100], with a diameter of 100 ± 0.4 mm and a thickness of 500 ± 10 μm. And the *N*-type GaAs ((0.8–9) × 10^–3^ Ω cm) was purchased from Zhejiang Lijing Photoelectric Technology Co., LTD, with a specification of 2 inches. The crystal was planar oriented along [100], with a diameter of 50.8 ± 0.1 mm and a thickness of 350 ± 10 μm. (2) Denton magnetron Sputtering coating apparatus (Discovery 635) was used to sputter about 0.85 μm gold film on the back of the semiconductor as the back electrode. (3) Use a glass knife to cut 2 cm × 3 cm semiconductor polishing wafer for friction with the metal slider.

### Fabrication of the TVNG with Interfacial Lubrication

By adding paraffin oil (the paraffin oil was purchased from Aladdin www.aladdin-e.com), DI and Mxene solution (the Mxene solution was purchased from Foshan Xinene Technology Co., LTD) to the friction interface of Cu sliders and semiconductors, the TVNG with interfacial lubrication was prepared.

### Measurement

Including TVNG performance test and self-powered pressure sensor test. The sliding process was conducted by a linear motor (TSMV120-1S). The short-circuit current, open-circuit voltage, and transferred charges of the TVNG was measured by a programmable electrometer (Keithley model 6514), the test data in this manuscript adopts absolute value. The atomic force microscope (AFM) image of the *P*-type Si, *N*-GaAs and Cu were measured by atomic force microscope test (produced by Brock GMBH, Germany). The Raman spectra of the *P*-type Si and the MXene were measured by confocal micro-Raman spectroscopic instrument (LABRAM HR EVOLUTION). The X-ray diffraction (XRD) of the MXene was measured by XRD powder test equipment (Xpert3 Powder). The electrical conductivity of lubricants was measured by four-point probe technique (HQ40D-Multi). The 2D and 3D optical surface profiler images were measured by three-dimensional topography profilometer (GT-X). The energy spectrum test was measured by field emission scanning electron microscope (X-MAX). The scanning electron microscope (SEM) images were measured by cold field scanning electron microscopy (SU8020). The dynamic resistance of TVNGs was measured by digital multimeter (VICTOR VC9808^+^) with multiple measuring when the TVNG is at working state.

## Results and Discussion

### Structure and Performance of TVNG with Interface Lubricant

**Fig. 1:**
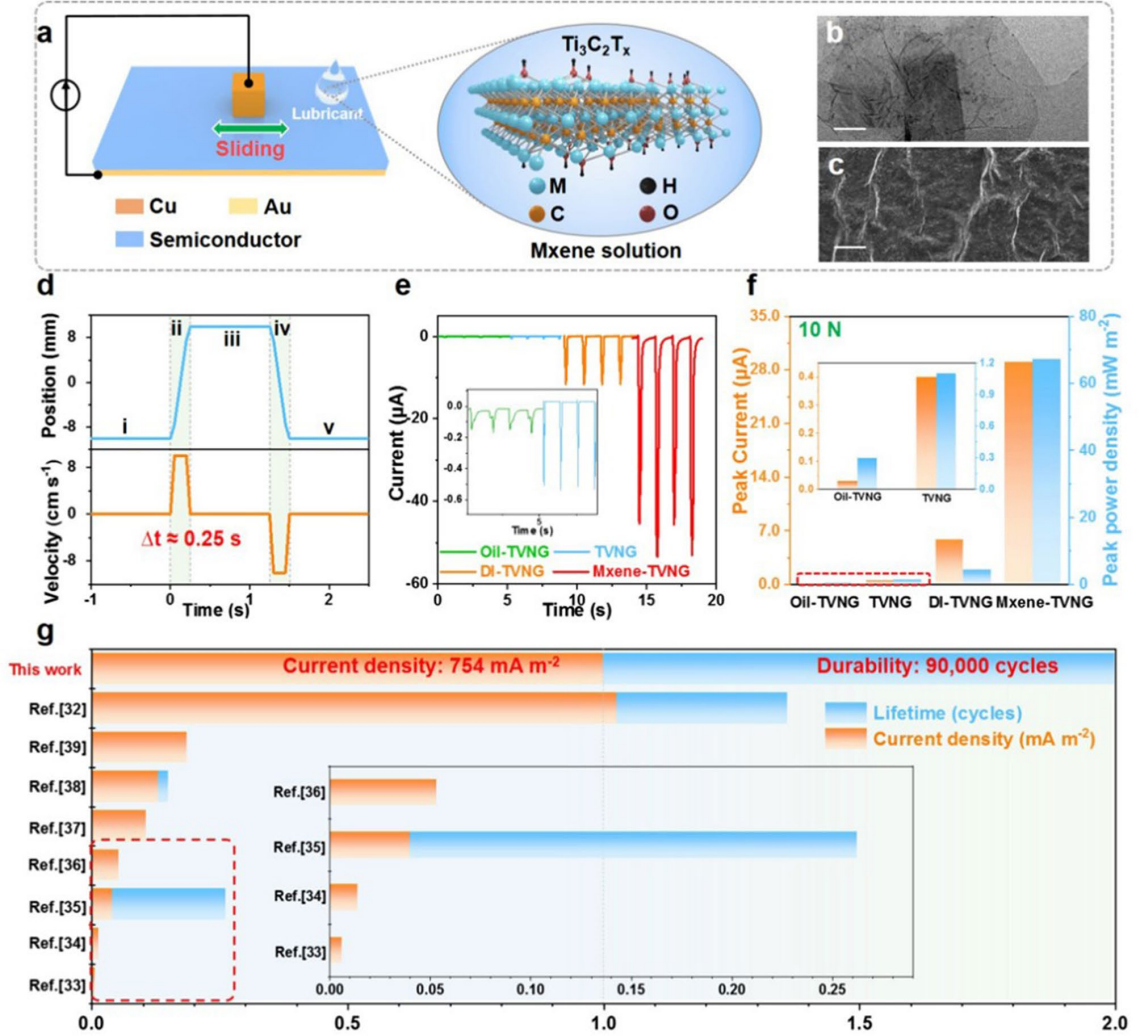
Structure and performance of TVNG with interface lubricant. **a** 3D structure of TVNG and its external circuit connection diagram. The illustration is the structural formula of Ti_3_C_2_Ti_*x*_ Mxene, which is the main component of Mxene solution lubricant. **b** TEM image of the MXene. **c** SEM image of the MXene. **d** The relative position and velocity of the Cu slider and the semiconductor wafer as a function of time. **e** Compare the output of short-circuit current in TVNG interface with different lubricants, the amount of lubricants is 5 μL and the applied pressure is 10 N (experiment condition: velocity 0.1 m s^−1^, displacement 20 mm). **f** The maximum peak power density and corresponding short-circuit current under the matched impedance (experiment condition: pressure 10 N, displacement 20 mm, 5 μL lubricate). **g** Comparison of the circuit density and lifetime with reported different type macro TVNGs based on the tribovoltaic effect

The 3D structure and external circuit diagram of TVNG with lubrication are shown in Fig. 1a, where copper and semiconductor wafer are used as friction pairs. Lubricant is added to the friction interface to form a lubricated TVNG, and the enlarged illustration is the structural formula of Ti_3_C_2_T_*x*_ MXene aqueous solution, where M and T_*x*_ represents the transition metal atom of Ti_3_ and surface groups, respectively. From the transmission electron microscopy (TEM) image (Fig. 1b) and the SEM image of MXene (Fig. 1c), it can be seen that MXene has two-dimensional layered fold structure, which has a certain lubrication characteristic [40–43]. The relative position and velocity of the Cu as function of time are recorded in Fig. 1d, and the corresponding equivalent circuit diagram is displayed in Fig. S1. From the I–V curve of TVNG at different pressures (Fig. S2), better performance can be obtained by using higher applied force. Therefore, the test pressure in Fig. S3 is fixed at 10 N to explore the I–V curve of TVNG lubricated by multiple liquids. It can be seen from Fig. S3 that all I–V curves of different lubricated TVNGs show exponential increase trend with the increase of the bias voltage, indicating that interface lubrication of TVNGs still have Schottky contact. In addition, MXene solution lubricated TVNG (MXene-TVNG) exhibits the best output performance, followed by deionized water (DI) lubricated TVNG (DI-TVNG). It is noteworthy that oil lubricated TVNG (Oil-TVNG) has a lower current output compared to original device. These results indicate that both MXene solution and DI are beneficial to improving the current output of TVNG, while oil plays an opposite role in the current output of TVNG.

To better understand the effect of interface lubricant on the output performance of TVNG, the short-circuit current and open-circuit voltage of four kinds of TVNG were investigated. The short-circuit current and open-circuit voltage of TVNGs at different pressures are shown in Figs. 1e and S4. With the increase of applied force, the voltage of TVNGs almost keeps constant value, whereas the corresponding currents improves (Fig. S4(a, c, e, g)). Under 10 N, the highest values of 53.49 µA is obtained by MXene-TVNG, which is 4.4 times of DI-TVNG with 12 µA, 104.8 times of original TVNG with 0.51 µA, and 314.6 times of oil-TVNG with 0.17 µA (Fig. 1e). Besides, the peak current and peak power density (PPD) of TVNGs under different loads were also measured as exhibiting in Figs. 1f and S4(b, d, f, h). Clearly, the maximum PPDs of TVNG without lubrication and lubricated by oil, DI and MXene solution are 1.08, 0.265, 4.04, and 67.19 mW m^−2^ with matching resistance 0.7 MΩ, 7 MΩ, 3 KΩ, and 2 KΩ respectively. The results further demonstrate the optimum enhancement of MXene solution in the output performance of TVNG.

Figure 1g shows the comparison of current density and lifetime with reported different type macro sliding-mode metal–semiconductor TVNGs. Clearly, a high current density value of 754 mA m^−2^ was achieved in this work, which is similar to our previous work of 775 mA m^−2^ [32]. Excitingly, the lifetime of TVNG is prolonged to 90,000 cycles and the output also remains about 90% of the initial value in this work, which is improved by three-fold compared to the reported highest value of lifetime (30,000 cycles) [32].

### Effects of Different Interface Lubricants on Output Performance of TVNGs (*P*-Type Si and Cu)

**Fig. 2:**
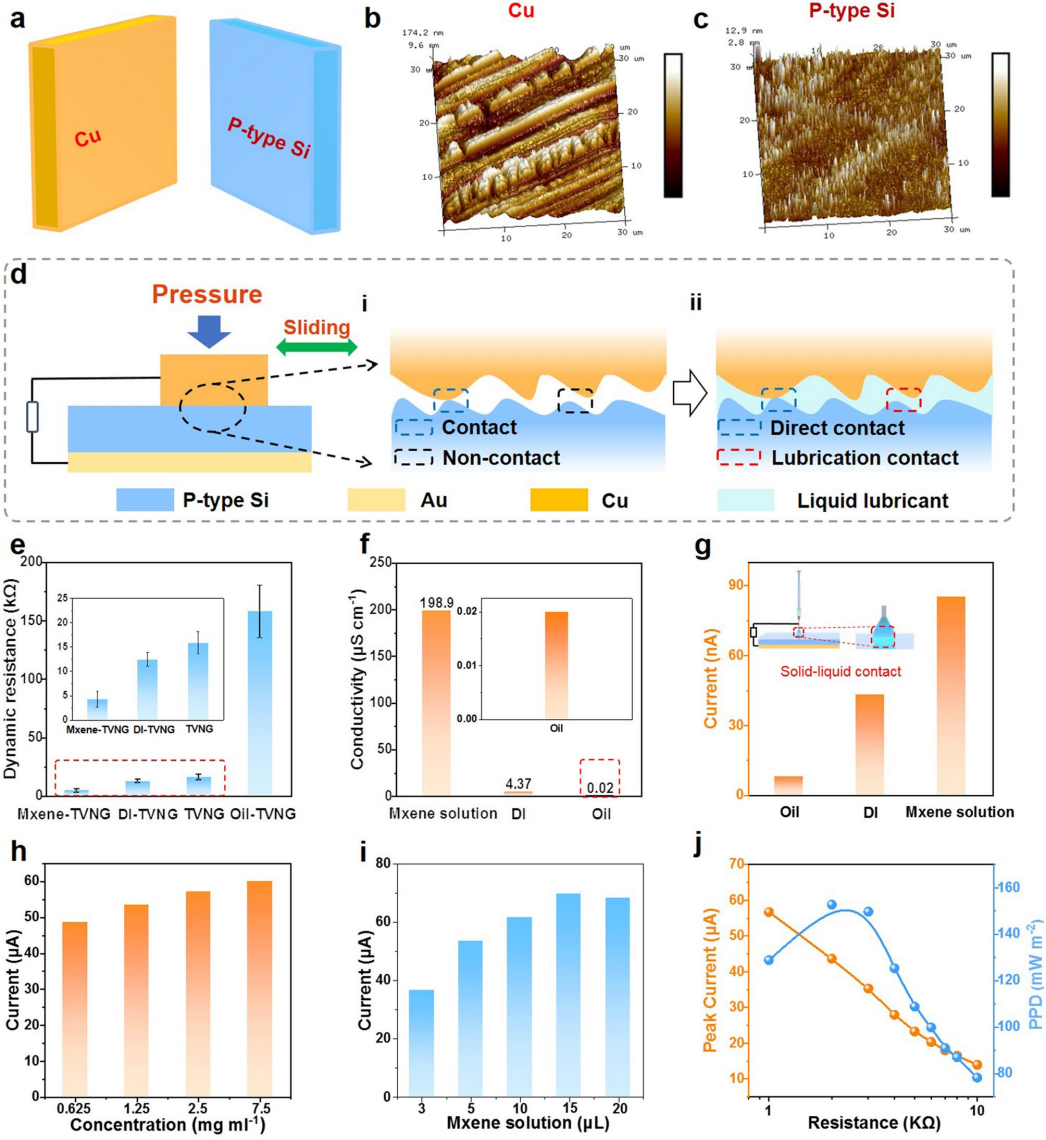
Effects of different interface lubricants on output performance of TVNGs (*P*-type Si and Cu). **a** Friction material pairs are Cu and *P*-type Si. **b** AFM image of the Cu film. **c** AFM image of the *P*-type Si. **d** The planar structure and microstructure of TVNG. **i** Interfacial microstructure of TVNG without lubricant. **ii** Interfacial microstructure of MXene-TVNG. **e** Dynamic resistance of oil-TVNG, original TVNG, DI-TVNG and MXene-TVNG (experiment condition: pressure 10 N, motion displacement 20 mm, velocity 0.1 m s^−1^). **f** Electrical conductivity of lubricants. **g** Comparing the short-circuit current generated by sliding friction between different lubricants and *P*-type Si, the distance between syringe needle and *P*-type Si is about 1 mm (experiment condition: velocity 0.1 m s^−1^, displacement 20 mm). **h** Short-circuit current under different concentration of MXene solution (experiment condition: pressure 10 N, velocity 0.1 m s^−1^, displacement 20 mm, 5 μL Mxene solution). **i** Short-circuit current under different amount of MXene solution (experiment condition: pressure 10 N, velocity 0.1 m s^−1^, displacement 20 mm, 1.25 mg mL^−1^ MXene solution). **j** The peak current and PPD of MXene-TVNG under 10 N, 10 µL MXene solution as an interfacial lubricant (experiment condition: pressure 10 N, motion displacement 20 mm, velocity 0.2 m s^−1^)

The effects of different interface lubricants on the output performance of TVNGs were investigated from a microscopic perspective. Cu and *P*-type Si are the material pairs of TVNGs (Fig. 2a). Figure 2b, c shows the AFM image of the unused Cu and *P*-type Si, it can be seen that the *P*-type Si surface and Cu surface are convex and concave on the microscopic scale, thus plenty of gap will exist in the friction interface. When Cu slides on the *P*-type Si surface, complete contact is difficult to achieve because of the existence of gaps, leading to a lower electron transport efficiency. If a liquid fluidity with well polar liquid is employed, the liquid lubricant can fill the gap, so as to improve the effective area of the contact transfer electron hole pairs. Figure S6 shows the charges transfer diagram of original TVNG at the state of static, dynamic, and after friction in a period time. In the initial contact state, no current flows through the external circuit (Fig. S5a). When Cu rubs against with semiconductor, the output performance remains stable due to the full contact of the friction material (Fig. S5b). After a period of friction, serious wear will improve the surface roughness, accompanied with insufficient contact with Cu and semiconductor, causing rapid attenuation of output performance (Figs. S5c and S6). According to the above analyses, the micro-interface of TVNG is illustrated in Fig. 2d, where direct contact and non-contact coexist at the micro-interface of original TVNG (Fig. 2di). By adding liquid solution at the interface, the unsmooth surface is filled out to achieve direct contact and lubrication contact Fig. 2dii). To reveal the role of different lubricants in TVNG, the dynamic resistances of MXene-TVNG, DI-TVNG, original TVNG and oil-TVNG are measured, which are about 4.21, 12.4, 15.79 and 157.72 kΩ, respectively (Fig. 2e). The difference of dynamic resistances may result from the different electrical conductivity of MXene solution, DI and oil, which are 198.9, 4.37, and 0.02 μS cm^−1^, respectively (Fig. 2f). Specifically, when high conductive liquid fills the gap in the interface, dynamic resistance of TVNG will decrease compared to original device, and then more electron–hole pairs can pass through under the same built-in electric field, thus improve the output performance of TVNG. Furthermore, considering the solid–liquid tribovoltaic effect in liquid lubricated TVNG, different lubricants are placed in a syringe to contact with *P*-type Si (Fig. 2g). With sliding on *P*-type Si, the short-circuit currents of MXene solution, DI and oil are 85.23, 43.23 and 8 nA, respectively (Fig. 2g). The results indicate that polarity of liquid solution is a key factor to achieve solid–liquid tribovoltaic effect, and more carriers can be generated by increasing the polarity of solution. Compared to the current density and charges density of original TVNG with 50.5 mA m^−2^ and 0.67 mC m^−2^, the values of MXene-TVNG increases by 12.2 times to 617.4 mA m^−2^ and 118.3 times to 79.31 mC m^−2^ (Fig. S7), which demonstrate the enhancement performance by using MXene solution. The influence of motion parameters on the output of TVNG was also investigated. As shown in Fig. S8, with the increase of the velocity from 0.01 to 0.2 m s^−1^, the short-circuit current and transferred charges increase from 7.725 to 75.422 μA and from 4.614 to 8.335 μC, respectively. Besides, the current output is also improved with the elevating of displacement and acceleration velocity (Fig. S9). With increasing the concentration (from 0.675 to 7.5 mg mL^−1^) and amount of MXene solution (from 3 to 20 μL), the current output can be enhanced firstly and then tend to be stable (Fig. 2h, i), which contribute to larger conductivity (Fig. S10) of MXene solution with increasing the concentration and better contact efficiency in the interface with the rising MXene solution amount. As shown in Fig. 2i, the current output increases with the addition of MXene solution, however, when the addition of MXene solution increases to 20 μL, the current output do not increase, which may contribute to the lower contact efficiency of the interface induced by much MXene flakes at high amount of MXene solution. Besides, MXene-TVNG has a low matched impedance of 2 kΩ, where its peak current is 43.70 μA and maximum PPD is 152.8 mW m^−2^ (Fig. 2j). Furthermore, polar liquid interface lubrication strategy mentioned in this work has the same effectiveness in rotating TVNG (Note S1 and Fig. S11).

### Working Mechanism of Liquid Lubricated TVNG (*P*-Type Si and Cu)

**Fig. 3:**
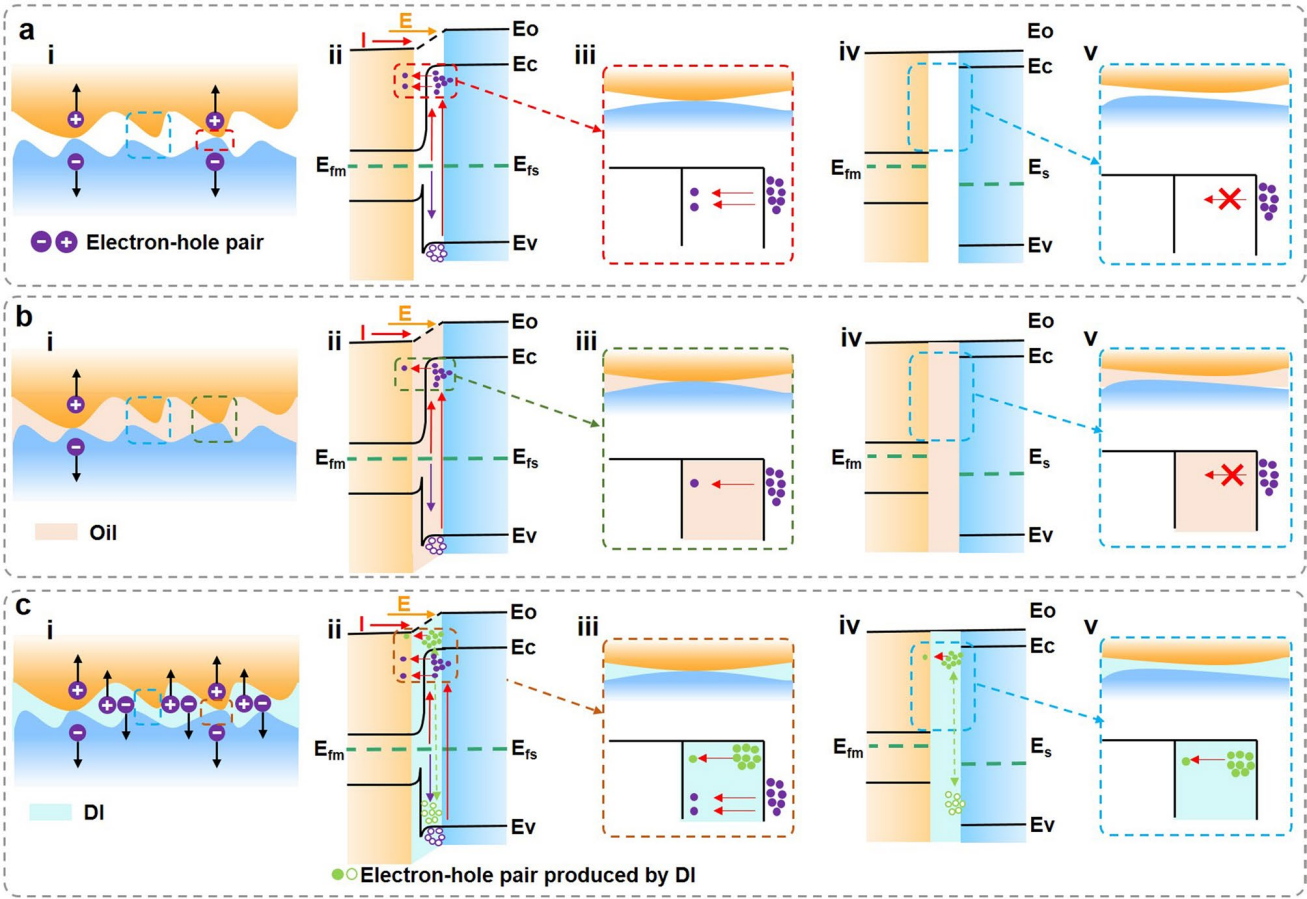
Working mechanism of liquid lubricated TVNG (*P*-type Si and Cu). **a** Interfacial electron–hole pairs transfer and energy band diagram of TVNG without lubricant. **b** Interfacial electron–hole pairs transfer and energy band diagram of oil-TVNG. **c** Interfacial electron–hole pairs transfer and energy band diagram of MXene-TVNG

Figure 3a–c present the electron–hole pairs transfer and energy band diagram of original TVNG, oil-TVNG and DI-TVNG, respectively. Figure S12a shows the energy band diagram of TVNG when Cu and *P*-type Si are in non-contact state. Cu contact with *P*-type Si (the work function of *P*-Si and Cu are about 4.911 and 4.65 eV [37]), the Cu and *P*-type Si remain relatively stationary, at static state there is no energy release and therefore no external current output (Fig. S12b).

The work function (*W*_Si_) of *P*-type Si is higher than that of (*W*_*m*_) Cu, and according to formula (1), the fermi energy level (*E*_f_) of *P*-type Si is smaller than that of Cu (*E*_0_ is the vacuum level).

1 $$E_{{\text{f}}} = E_{0} - W$$

When *P*-type Si is in close contact with Cu under pressure, the equilibrium of the system will be re-established. Due to the higher work function of *P*-type Si, electrons flow from Cu to *P*-type Si, and *P*-type Si is negatively charged on the surface, forming an internal electric field directed by Cu to *P*-type Si. In the dynamic state (Fig. 3a), there are two ways to excite electron hole pairs. Firstly, friction creates the “bindington” that excites electron–hole pairs, which drift under the action of built-in electric field (Fig. 3aii). Secondly, some surface electrons jump to higher energy levels under “bindington” excitation (Fig. 3aii). The transfer of electron hole pairs occurs at the interface where Cu and Si are direct contact (Fig. 3ai–iii), while never happen in non-contact interface (Fig. 3ai, iv and v). As for non-polar lubricated TVNG such as oil-TVNG (Fig. 3b), less electron–hole pairs transfer in the direct contact interface (Fig. 3bi–iii) and no electron–hole pairs occur in non-contact interface (Fig. 3bi, iv and v) because of the lower electrical conductivity of oil and higher dynamic resistance of oil, thus the performance of TVNG greatly reduces by oil lubricating. As for polar lubricated TVNG such as DI-TVNG, more charges carriers can be supplied to the interface due to solid–liquid tribovoltaic effect, accompanied with high electrical conductivity and low dynamic resistance, the transfer efficiency of electron hole pairs can be improved in both surface of direct contact (Fig. 3ci–iii) and lubricated contact (Fig. 3ci, iv and v), thus largely boosts the output performance of TVNG. In short, well polarity of liquid solution is a key parameter for achieve high performance TVNG.

### Stability of Liquid Lubricated TVNG (*P*-Type Si)

**Fig. 4:**
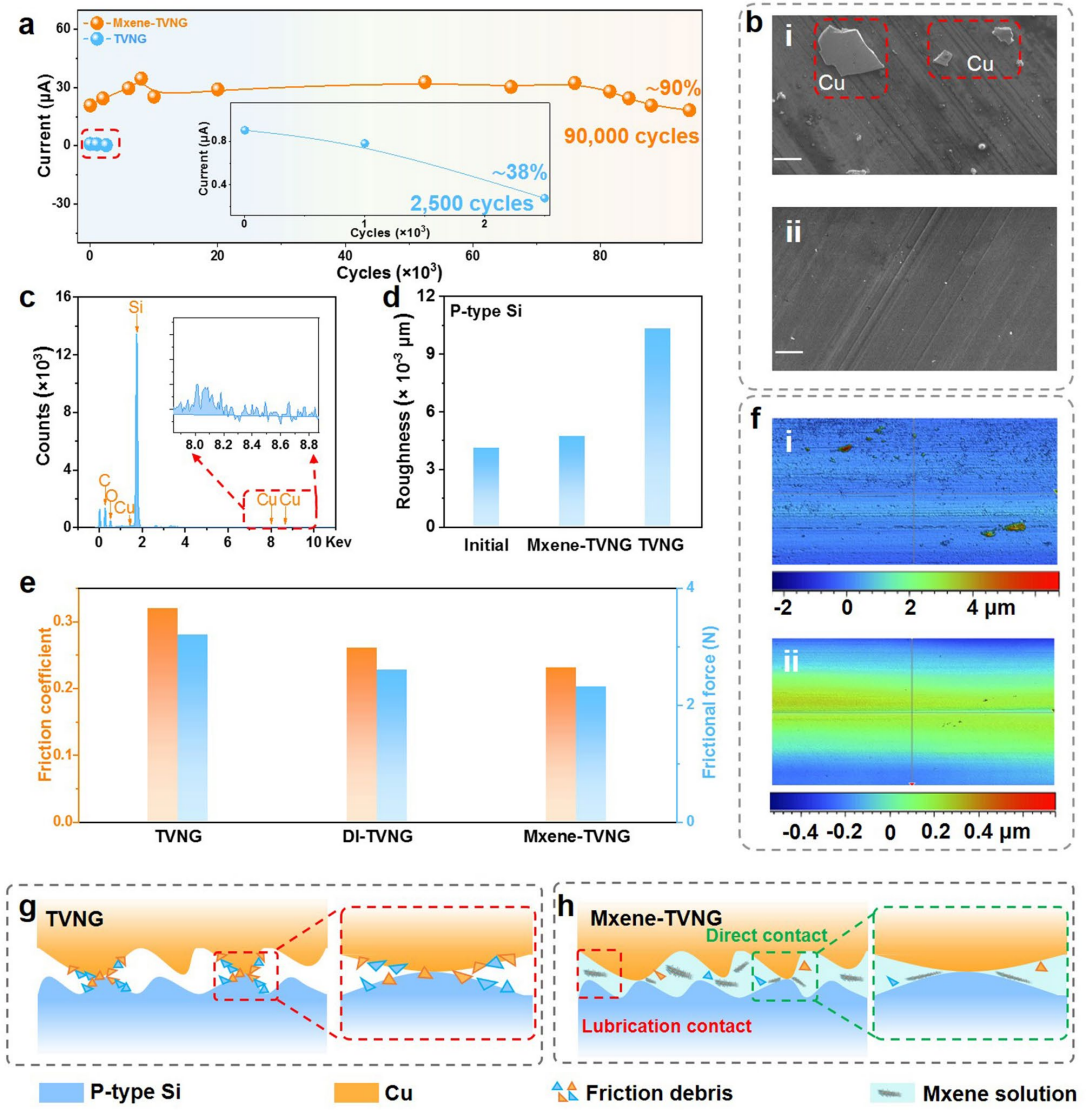
Stability of liquid lubricated TVNG (*P*-type Si). **a** The stability comparison of MXene-TVNG and original TVNG (experiment condition: pressure 10 N, motion displacement 10 mm, velocity 0.1 m s^−1^, Mxene solution 3 ml). **b** SEM images of *P*-type Si without lubricant after stability test **i**, and the SEM images of MXene-TVNG after stability test **ii** (Scale bar, 50 μm). **c** TVNG (*P*-type Si) without lubricant generates material transfer during friction and affects output stability, this is the energy spectrum of material transfer measured by the energy dispersion spectrometer. **d** Roughness comparison of the original silicon wafer, the MXene solution lubricated silicon wafer after 2,800 stability tests, and the silicon wafer without lubricant after 2,800 cycles.** e** The friction coefficient and friction force of lubricated TVNG (*P*-type Si) by different lubricants (experiment condition: pressure 10 N, motion displacement 20 mm, velocity 0.1 m s^−1^). **f** 2D optical surface profiler images of *P*-type Si without lubricant **i** after 90,000 stability tests, and the 2D optical surface profiler images of Mxene-TVNG **ii** after 90,000 stability tests. **g** The interface wear in TVNG without interface lubricant. **h** MXene solution is used as an interface lubricant to reduce interface wear

To investigate the influence of Mxene solution on the durability of MXene-TVNG (*P*-type Si), the physicochemical properties of Mxene were firstly measured. From the Raman spectrogram of MXene (Fig. S13a, b), the characteristic peaks of MXene appear, where the position of Peak D and Peak G are about 1339 and 1553 cm^−1^, respectively. Figure S13c is the XRD pattern of MXene, shifting the 002 peak towards a lower 2*θ* value shows expansion between the layers of MXene. From the TEM image (Fig. 1b) and the SEM image of MXene (Fig. 1c), it can be seen that MXene has two-dimensional layered fold structure which has a certain lubrication characteristic [40–43], which is benefit to reduce the interface wear and improve the lifetime of TVNG. Figure S6 shows the stability test of original TVNG, due to serious scratches and material transfer (Fig. 4bi, c), the current reduce gradually and then reverses after about 2,500 cycles, only maintaining about 38% current output of original value (Fig. 4a). The lifetime of TVNG can be improved by adding MXene solution as interface lubricant. To avoid the precipitate of MXene, we stir the solution to make it evenly disperse in the water before measuring the output performance of TVNG. In addition, during the sliding process, the MXene solution will have molecular motion along with the slider movement, which is also beneficial for the uniform dispersion of MXene solution (Note S2). As can be seen from Fig. 4a, MXene-TVNG can still maintain about 90% current output after 90,000 cycles, indicating that the MXene solution still exhibits well lubrication in TVNG after a long working interval. As shown in Fig. 4bii, there are only a few scratches in the surface of *P*-type Si after a long time of friction, indicating that MXene solution can reduce the interface wear and improve the service life of TVNG devices. Furthermore, the smaller roughness (Fig. 4d) of MXene solution lubricated silicon wafer and lower friction coefficient and friction force (Fig. 4e) of MXene solution lubricated TVNG than that of TVNG without lubrication further demonstrate the excellent interfacial lubricant effect of MXene solution. Besides, from the 2D and 3D optical surface profiler images (Figs. 4f and S14) after 90,000 stability tests, the wear height difference of *P*-type Si without lubrication is 6.751 μm, while the wear height difference of MXene solution lubricated *P*-type Si largely decreases to 0.74 μm, which further verify that Mxene solution with lubrication characteristics can anti-attrition and improve the lifetime of MXene-TVNG.

The mechanism of wear resistant by using MXene solution can briefly illustrate in Fig. 4g, h. As for TVNG, friction debris is generated at the direct contact position (The enlarged view shows the friction debris between the surface of Cu and Si in direct contact), and the wear debris continues to accelerate the interface wear resulting in a rapid decline in output (Fig. 4g). After adding MXene solution, direct contact and lubrication contact [40–45] will generate in the interface of TVNG (Fig. 4h), thus the produced debris can be removed from contact position to reduce wear and maintain a stable output for TVNG.

### Performance of MXene-TVNG (*N*-Type GaAs)

**Fig. 5:**
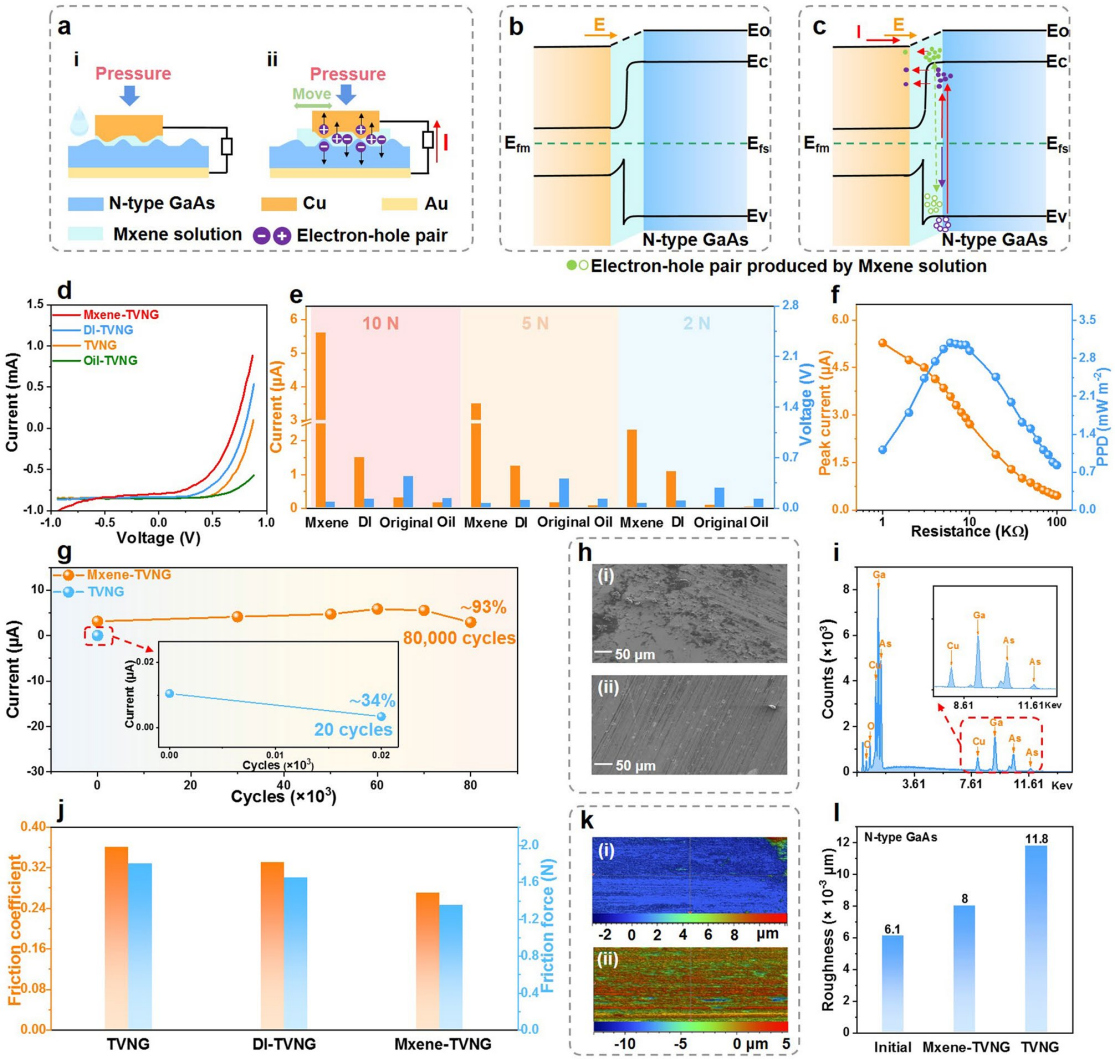
Performance of MXene-TVNG (*N*-type GaAs). **a** Working principle of the sliding TVNG (*N*-GaAs, Cu and Mxene solution). **b, c** Band structure diagram of the sliding MXene-TVNG (*N*-type GaAs).** d** *I–V* curves of TVNG (*N*-type GaAs) interface with different lubricants were compared, the amount of lubricants is 5 μL and the applied pressure is 10 N. **e** Compare the open-circuit voltage and short-circuit current of with different lubricants TVNG (*N*-type GaAs) under 10 N, 5 N, and 2 N pressure lubricants (experiment condition: lubricants 5 μL, velocity 0.1 m s^−1^, displacement 20 mm). **f** Peak current and PPD of MXene-TVNG (*N*-type GaAs) under different loads, 5 µL MXene solution as interfacial lubricant (experiment condition: pressure 10 N, displacement 20 mm, velocity 0.1 m s^−1^).** g** The stability test of MXene-TVNG (*N*-type GaAs) (experiment condition: pressure 10 N, motion displacement 10 mm, velocity 0.1 m s^−1^, Mxene solution 3 mL). **h** SEM images of *N*-type GaAs without lubricant after stability test **i**, and the SEM images of Mxene-TVNG (*N*-type GaAs) after stability test **ii** (Scale bar, 50 μm). **i** TVNG (*N*-type GaAs) without lubricant generates material transfer during friction and affects output stability. **j** Friction coefficient and friction force of lubricated TVNG (*N*-type GaAs) by lubricants (experiment condition: pressure 5 N, motion displacement 20 mm, velocity 0.1 m s^−1^). **k** 2D surface profiler images of *N*-type GaAs without lubricant **i** after 90,000 stability tests, and the 2D optical surface profiler images of MXene-TVNG **ii** after 90,000 stability tests. **l** The roughness comparison of the original *N*-GaAs, the Mxene solution lubricated *N*-GaAs after approximately 1100 cycles stability test, and the silicon wafer without lubricant after approximately 1100 cycles stability test

To illustrate the universality of MXene solution as lubricant for TVNG, we also explored the influence of MXene solution on the output performance and lifetime of direct band gap *N*-type GaAs and Cu. The working principle and band structure diagram of the sliding *N*-type GaAs–Cu based TVNG are shown in Fig. 5a–c, whose principle is similar to MXene-TVNG (*P*-type Si) (Fig. 3). From the AFM image, unsmooth structure is also observed on the surface of *N*-type GaAs (Fig. S15), thus the positive effect of interface lubrication may also take effect. Because of the better performance at higher applied force (Fig. S16), 10 N was used to explore the I–V curve of TVNG (*N*-GaAs) lubricated by different liquids. Apparently, MXene-TVNG has a higher current at the same bias voltage (Fig. 5d). Figure 5e shows the short-circuit current and open-circuit voltage output of original TVNG, oil-TVNG, DI-TVNG and MXene-TVNG at different pressure, respectively. As the pressure increases from 2 to 10 N, the corresponding short-circuit current increases from 0.0827 to 0.3012 μA, from 0.0196 to 0.154 μA, from 1.088 to 1.585 μA, and from 2.324 to 5.5 μA for original TVNG, oil-TVNG, DI-TVNG and MXene-TVNG, respectively. The open-circuit voltage difference of the four TVNGs is small. When pressure is 10 N, the short-circuit current of MXene-TVNG (*N*-GaAs) is 5.6 µA, which is 3.73 times of the DI-TVNG with 1.5 µA, 18.67 times of the original TVNG with 0.44 µA, and 37.33 times of oil-TVNG with 0.13 µA. The results further demonstrated the obvious enhancement of Mxene solution in the current output of TVNG. Besides, MXene-TVNG has a low matched impedance of 6 kΩ, where its peak current is 3.587 μA and maximum PPD is 3.088 mW m^−2^ (Fig. 5f).

The MXene solution as a lubricant can also improve the stability of MXene-TVNG (*N*-type GaAs). Due to serious scratches and material transfer (Figs. 5hi and i), the current reduce gradually and then reverses after about 20 cycles, only maintaining about 34% current output of original value (Figs. S17 and 5 g). After lubricated by MXene solution, the current output can maintain about 93% after 80,000 cycles (Fig. 5g). As shown in Fig. 5hii, there are only a few scratches in the surface of *N*-type GaAs after a long time of friction, indicating that MXene solution can reduce the interface wear and improve the service life of TVNG devices. Furthermore, the smaller roughness (Fig. 5l) of Mxene solution lubricated GaAs wafer and lower friction coefficient and friction force (Fig. 5j) of MXene solution lubricated TVNG than that of TVNG without lubrication further demonstrate the excellent interfacial lubricant effect of MXene solution. Besides, from the 2D and 3D optical surface profiler images (Figs. 5k and S18) after 80,000 stability tests, the wear height difference of *N*-type GaAs without lubrication is 12.17 μm, while the wear height difference of MXene solution lubricated *N*-type GaAs largely decreases to 4.87 μm, which further verify that MXene solution with lubrication characteristics can anti-attrition and improve the lifetime of MXene-TVNG (GaAs).

## Conclusion

In this work, MXene solution was proposed to lubricating TVNG, where a high value of 754 mA m^−2^ accompanied with a record durability of 90,000 cycles were achieved simultaneously. By contrasting multiple liquid lubricants (MXene solution, DI and oil), we found that polarity of lubricant is one of the key factors to improve the electric output performance of TVNG. Specifically, MXene solution with high polar and electrical conductivity largely reduces the dynamic resistance of TVNG, thus improves the output current density of TVNG. Besides, the additional charges carriers are verified by solid–liquid trobovoltic effect between polar liquid and *P*-type Si, which is proposed as the other factor to achieve high performance of lubricated TVNG. Compared with original TVNG and oil-TVNG, the current output of MXene-TVNG increase by 104.8 times and 314.6 times, respectively. As a typical two-dimensional material added into lubricant, MXene exhibits good lubricating property to reduce the mechanical wear of TVNG, thus the MXene-TVNG (*P*-type Si) can still maintain about 90% of current output after 90,000 cycles. Moreover, lubricating by MXene solution, the short-circuit current of Cu–*N*-type GaAs based TVNG also improves 18.67 times and 37.33 times compared to the original TVNG and oil-TVNG, and the MXene-TVNG (*N*-type GaAs) still maintain about 93% of current output after 80,000 cycles, which demonstrates the universality of MXene solution lubrication strategy. This work reveals the key factors of liquid lubricants in TVNG, and guides the design of high performance TVNG through liquid interface lubrication.

### Supplementary Information

Below is the link to the electronic supplementary material.

Supplementary file1 (PDF 975 KB)
