# Supplementary material for: MXene Lubricated Tribovoltaic Nanogenerator with High Current Output and Long Lifetime
Source: Nanomicro Lett. 2023 Oct 7;15:218. doi: 10.1007/s40820-023-01198-z (PMC10560292; doi:10.1007/s40820-023-01198-z)
Supplement: Supplementary file 1 — Supplementary file1 (PDF 975 KB) [file 40820_2023_1198_MOESM1_ESM.pdf]

Supporting Information for

MXene Lubricated Tribovoltaic Nanogenerator with High Current Output and Long Lifetime

Wenyan Qiao[1, 2, #], Linglin Zhou[1, 2, #], Zhihao Zhao[1, 2, #], Peiyuan Yang[1], Di Liu[1, 2], Xiaoru Liu[1, 2], Jiaqi Liu[1], Dongyang Liu[1], Zhong Lin Wang[1, 2, 3], Jie Wang[1, 2, *]

[1]Beijing Institute of Nanoenergy and Nanosystems, Chinese Academy of Sciences, Beijing 100083, P. R. China

[2] School of Nanoscience and Engineering, University of Chinese Academy of Sciences, Beijing 100049, P. R. China

[3] School of Materials Science and Engineering, Georgia Institute of Technology, Atlanta, GA 30332, USA

[#] Wenyan Qiao, Linglin Zhou, and Zhihao Zhao contributed equally to this work.

*Corresponding author. E-mail: wangjie@binn.cas.cn (J. Wang)

Supplementary Figures

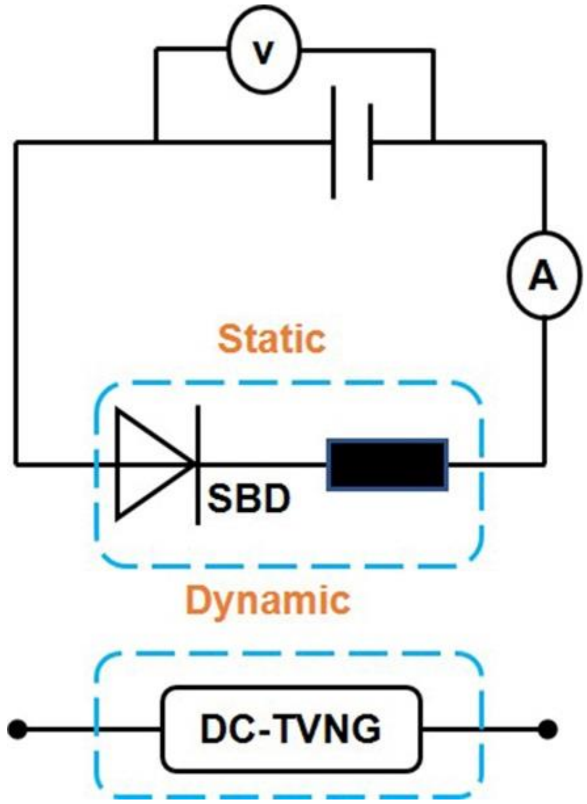

Fig. S1 TVNG equivalent circuit diagram under static and dynamic conditions

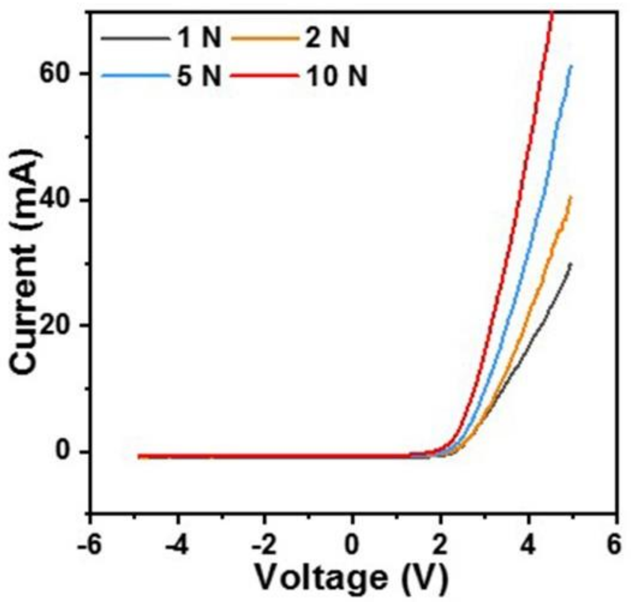

Fig. S2 I-V curve of TVNG (P-type Si and Cu) at different pressures (without adding lubricant at the interface)

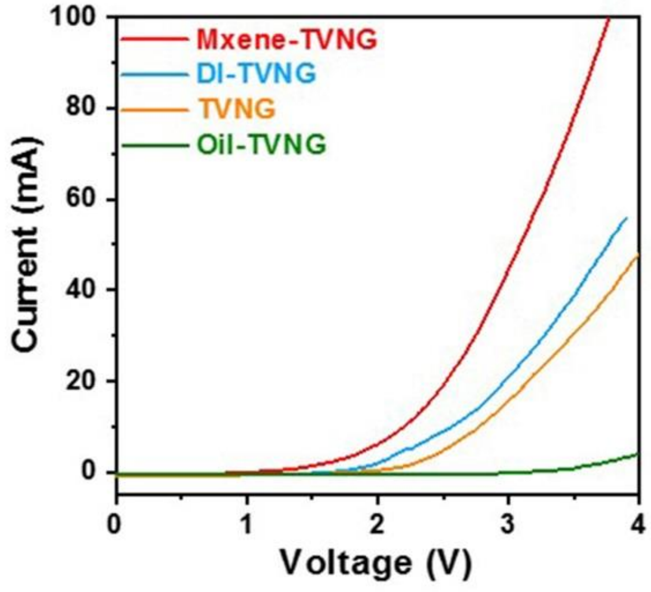

Fig. S3 I-V curve of TVNG (P-type Si and Cu). The I-V curves of TVNG interface with different lubricants were compared, the amount of lubricants is 5 μL and the applied pressure is 10 N

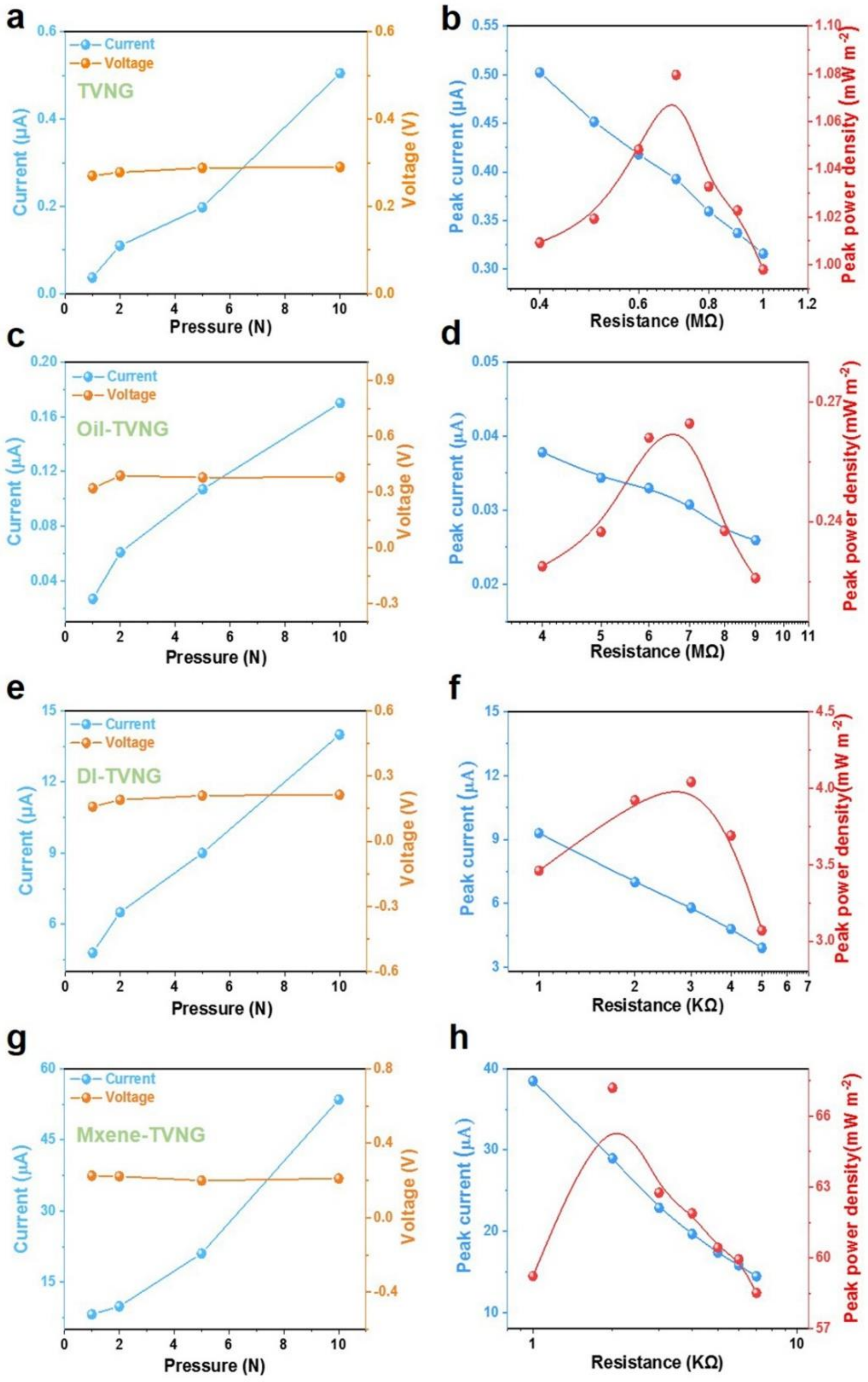

Fig. S4 a Short-circuit current and open-circuit voltage output of original TVNG at different pressure. **b** The peak current and PPD of original TVNG under different loads. **c** Short-circuit current and open-circuit voltage output of paraffin oil-TVNG at different pressure (5 μL paraffin oil). **d** The peak current and PPD of paraffin oil-TVNG under different loads. **e** Short-circuit current and open-circuit voltage output of DI-TVNG at different pressure (5 μL DI). **f** The peak current and PPD of DI-TVNG under different loads. **g** Short-circuit current and open-circuit voltage output of MXene-TVNG at different pressure (5 μL MXene solution). **h** The peak current and PPD of MXene-TVNG under different loads

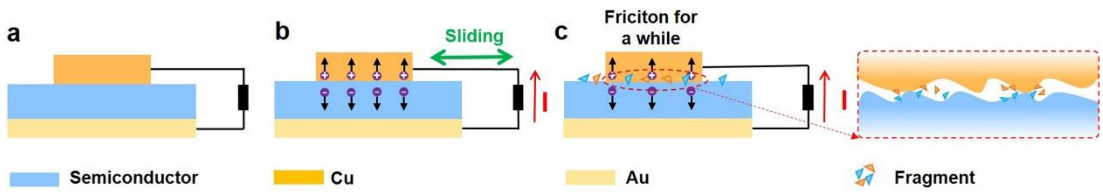

Fig. S5 a In the initial contact state, no current flows through the external circuit. **b** In the initial friction state, the output performance remains stable due to the full contact of the friction material. **c** After a period of friction, insufficient contact leads to reduced output

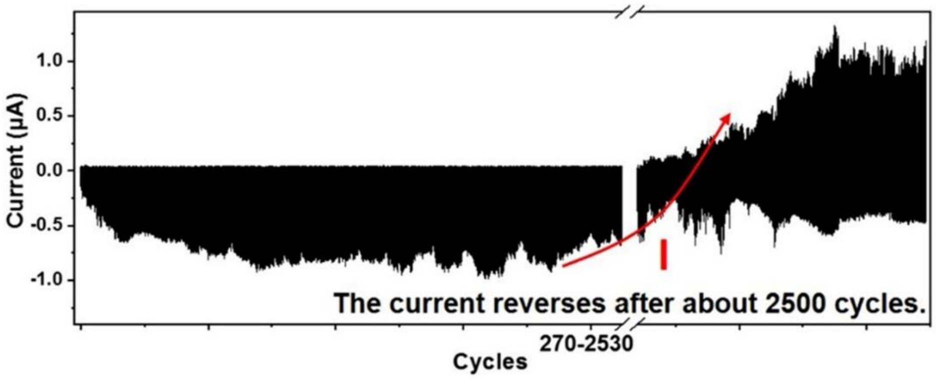

Fig. S6 The stability test of original TVNG (P-type Si)

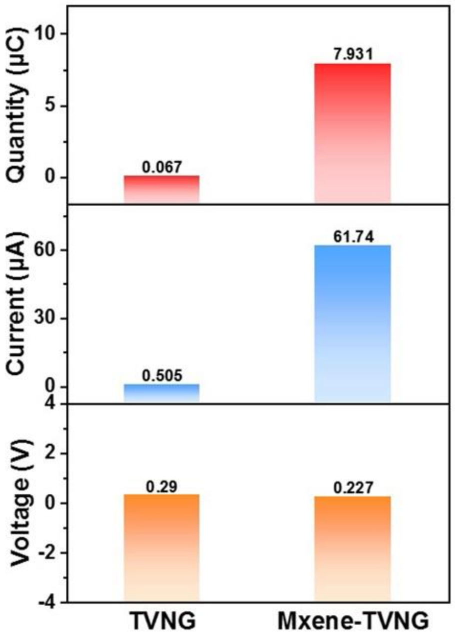

Fig. S7 Comparison of electric charges, short-circuit current and open-circuit voltage of original TVNG and MXene-TVNG (10 μL MXene solution)

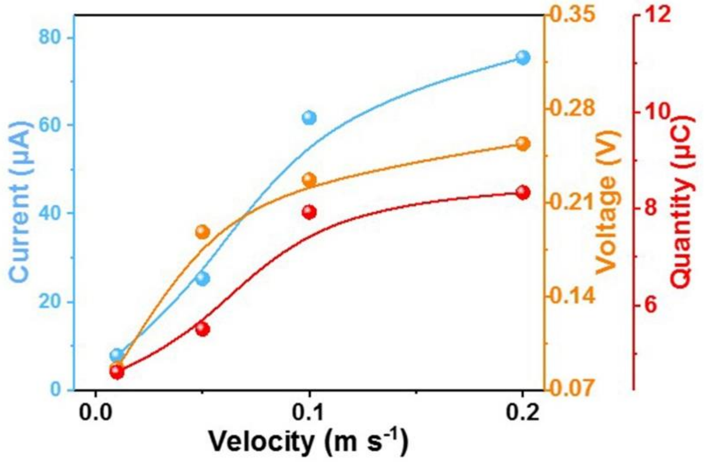

Fig. S8 The open-ircuit voltage, short-circuit current and transfer charges of MXene-TVNG at different velocity, 10 μL MXene solution as an interfacial lubricant (experiment condition: pressure 10 N, displacement 20 mm)

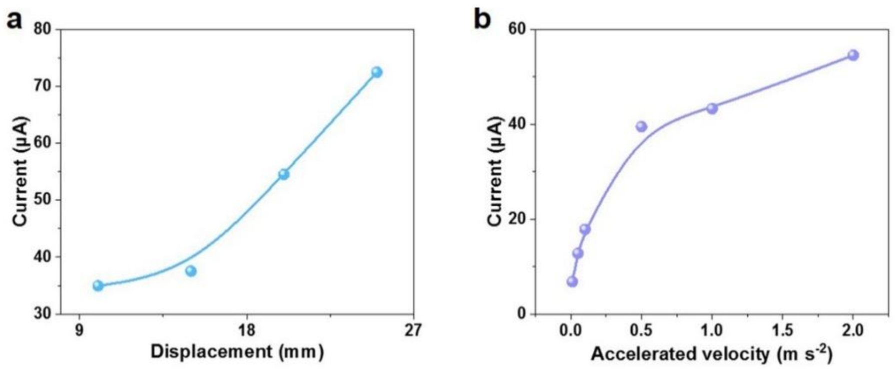

Fig. S9 a The short-circuit current of MXene-TVNG at different displacements (experiment conditions: pressure 10 N, velocity 0.1 m s^{-1}, MXene solution 5 μL). **b** The short-circuit current of MXene-TVNG at different accelerated velocity (experiment conditions: pressure 10 N, velocity 0.1 m s^{-1}, displacement 20 mm, MXene solution 10 μL)

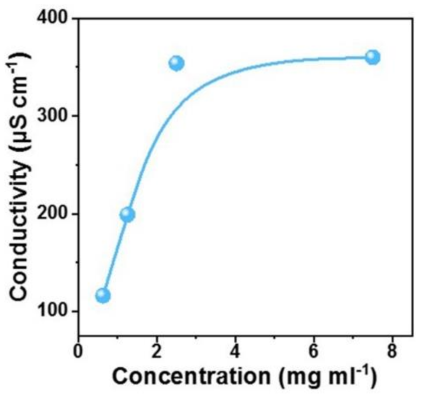

Fig. S10 The electrical conductivity under different concentration of MXene solution (experiment condition: pressure 10 N, velocity 0.1 m s^{-1}, displacement 20 mm, 5 μL MXene solution)

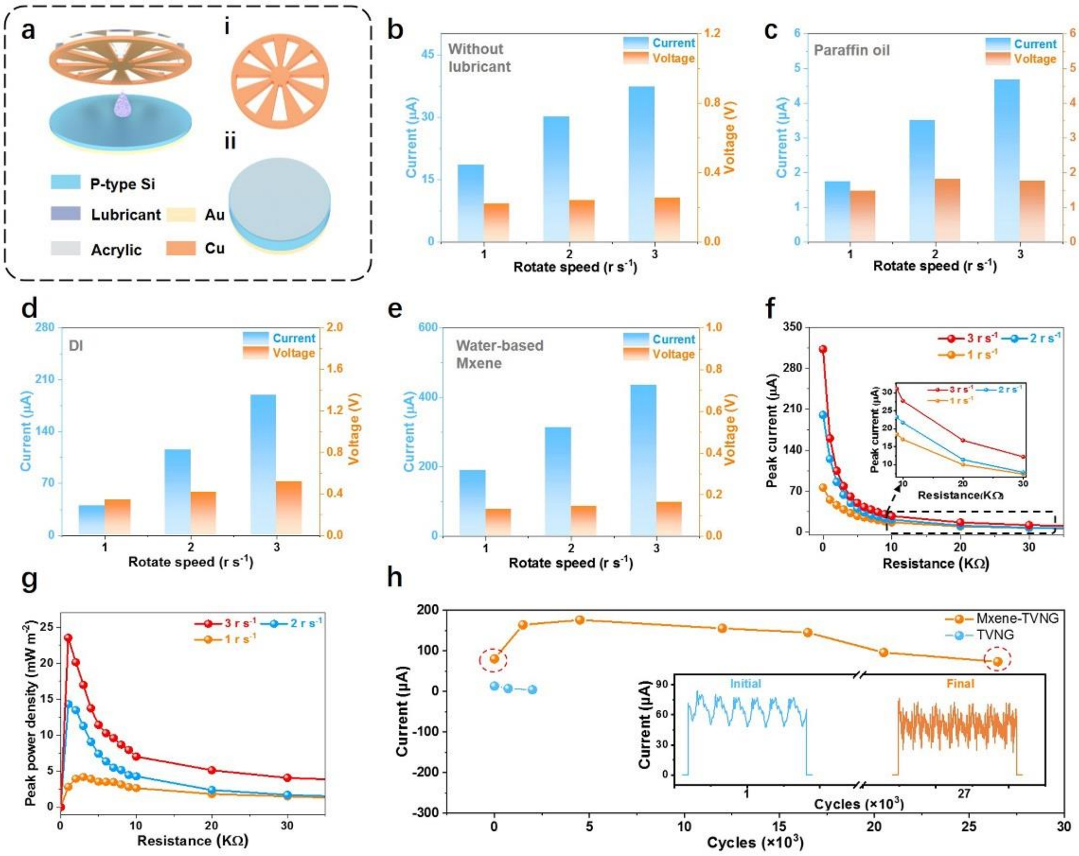

Fig. S11 Output performance of rotary TVNGs with interface lubricants. **a** The 3D structure of rotary TVNG. **b** The cu rotates on the P-type Si without adding lubricant at the interface (TVNG). The rotary TVNG's short-circuit current and open-circuit voltage output at different rotate speeds. **c** The cu rotates on the P-type Si, and paraffin oil is added as lubricant at the interface (oil-TVNG). The rotary oil-TVNG's short-circuit current and open-circuit voltage output at different rotate speeds. **d** The Cu rotates on the P-type Si, and DI is added as lubricant at the interface (DI-TVNG). The rotary DI-TVNG's short-circuit current and open-circuit voltage output at different rotate speeds. **e** The cu rotates on the P-type Si, and MXene solution is added as lubricant at the interface (MXene-TVNG). The rotary MXene-TVNG's short-circuit current and open-circuit voltage output at different rotate speeds. **f** The peak current output of rotary MXene-TVNG under external load. **g** The peak power density of rotary MXene-TVNG under external load. **h** The stability test of rotary TVNG and MXene-TVNG

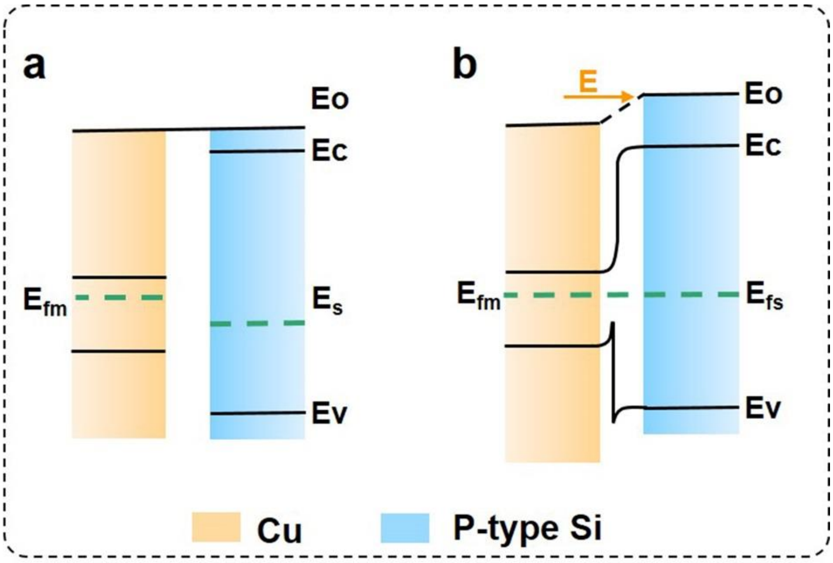

Fig. S12 The energy band diagram of TVNG at static state. **a** Cu and P-type Si are in non-contact state. **b** Cu and P-type Si are in contact state

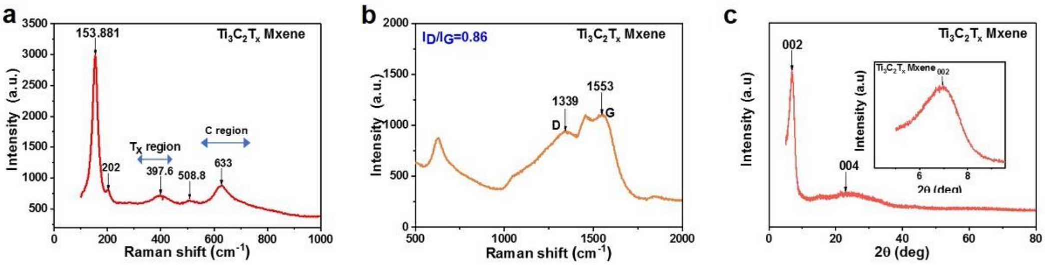

Fig. S13 **a** Raman spectra of the MXene. **b** Raman spectra of the G and D peaks of MXene. **c** XRD of the MXene

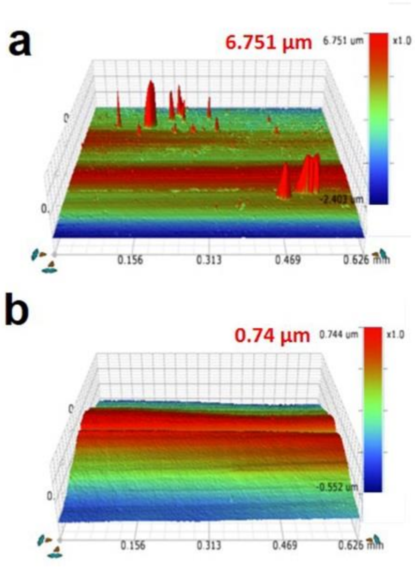

Fig. S14 3D optical surface profiler images of P-type Si without lubricant **i** after 90,000 stability tests, and the 3D optical surface profiler images of MXene-TVNG **ii** after 90,000 stability tests

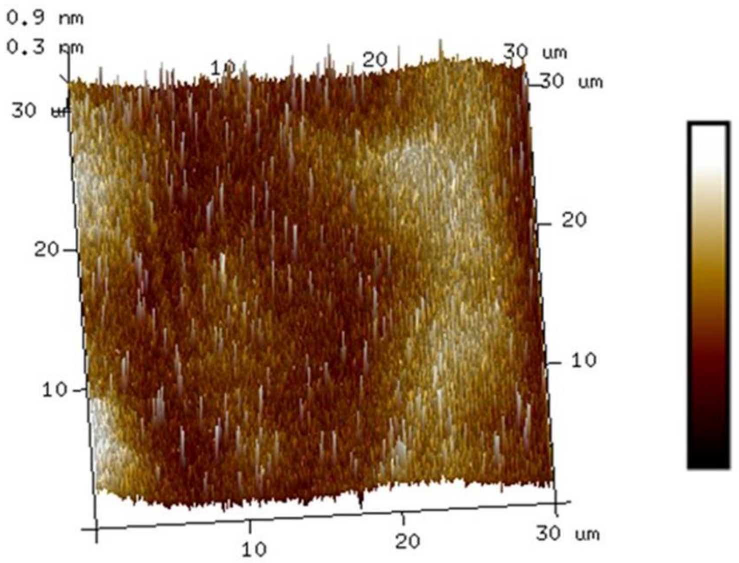

Fig. S15 AFM image of the N-type GaAs

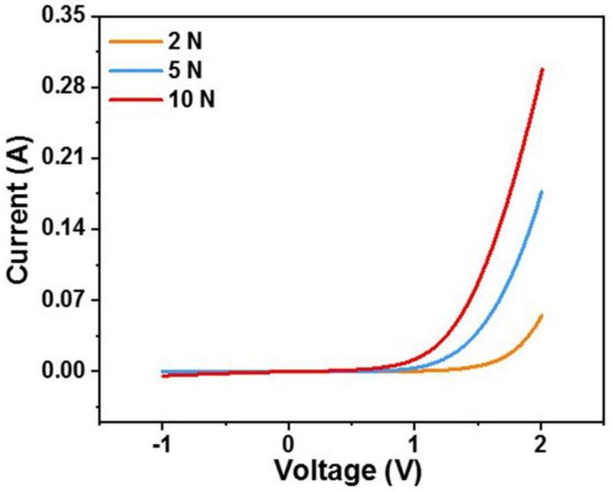

Fig. S16 I-V curve of without lubricated TVNG (N-type GaAs) at different pressures

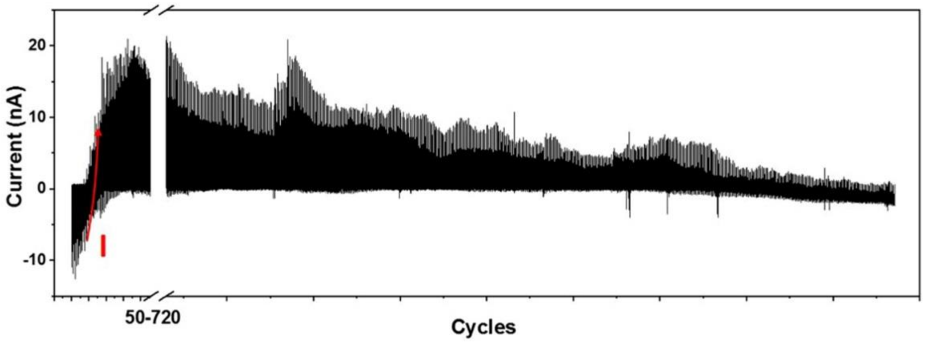

Fig. S17 The stability test of TVNG (GaAs) without lubricant

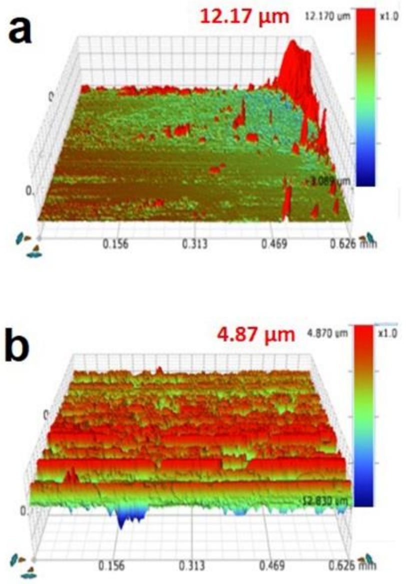

Fig. S18 3D optical surface profiler images of N-type GaAs without lubricant **i** after 90,000 stability tests, and the 3D optical surface profiler images of MXene-TVNG **ii** after 90,000 stability tests

Note S1 The polar liquid interface lubrication strategy mention in this manuscript has the same effectiveness in rotating TVNG

Compared with linear motion, rotational motion is more convenient to operate, hence, we also explore the performance of rotary TVNGs with interface lubricants. The 3D structure diagram of rotary TVNG is shown in Fig. S11a, where mainly consists of a rotor and a stator, and different liquids were added to the interface as lubricants. Simplify, the rotor of TVNG is mainly consists of Cu electrode (Fig. S11a(i)), and the stator is composed of P-type Si (Fig. S11a(i)). Figure S11b-e show the short-circuit current output and open-circuit voltage output of TVNGs without interface lubricant (TVNG), paraffin oil lubricated TVNG (oil-TVNG), DI lubricated TVNG (DI-TVNG), and MXene solution lubricated TVNG (MXene-TVNG), respectively. It can be seen that the short-circuit currents of all four TVNGs increase with the increase of rotate speed, but open-circuit voltages have a litter change. When the rotate speed is 3 r s^{-1}, the short circuit currents of TVNG without interface lubricant, oil-TVNG, DI-TVNG, and MXene-TVNG are 37.198, 4.667, 189.17, and 434.65 μA, respectively. Apparently, MXene-TVNG has a better output performance compared with the other interface lubricated rotary TVNGs. Fig. S11f and Fig. S11g show the peak current and peak power density (PPD) of MXene-TVNG at different rotation speeds along with the external loads. Clearly, greater values of peak current and PPD can be obtained at larger rotation speed. Specifically, MXene-TVNG has low-impedances of 1, 1, and 3 kΩ at 3, 2 and 1 r s^{-1}, where the corresponding peak currents are 160.498, 125.24, and 39.12 μA and maximum PPDs are 23.54, 14.33, and 4.19 mW m^{-2}, respectively. After 26,500 cycles, the MXene-TVNG still maintains 91% current output compared with initial value of 80.44 μA (Fig. S11h). However, the TVNG still maintains 32.2% current output compared with initial value of 13.18 μA after 2,000 cycles. Hence, MXene

solution as an interface lubricant for rotating TVNG still has the effect of improving the current output and lifetime simultaneously. In addition, the lifetime of rotating TVNG is not as long as that of linear TVNG. The possible reason is that with the addition of 3 ml MXene solution, the interface contact area of rotating TVNG (diameter 3.8 cm) is larger and the consumption of interface lubricant is faster. To sum up, polar liquid interface lubrication strategy mention in this manuscript has the same effectiveness in rotating TVNG.

Note S2 The effect of dispersity on lubricity of MXene solution, the ways to avoid MXene agglomeration

Good dispersion can prolong the effectiveness of MXene solution. If the MXene solution is agglomerated, the lubricity will be weakened and the lifetime of TVNG will be reduced. In this work, to avoid the precipitate of MXene, we stir the solution to make it evenly disperse in the water before measuring the output performance of TVNG. In addition, during the sliding process, the MXene solution will have molecular motion along with the slider movement, which is also beneficial for the uniform dispersion of MXene solution.
